# Poly[[diaqua-μ_2_-hydroxido-(μ_7_-2-phos­pho­nato­ethane­sulfonato)­dicopper(II)] trihydrate]

**DOI:** 10.1107/S1600536808033229

**Published:** 2008-10-18

**Authors:** Andreas Sonnauer, Alexandra Lieb, Norbert Stock

**Affiliations:** a Institute of Inorganic Chemistry, Christian-Albrechts-University, Max-Eyth-Strasse 2, D 24118 Kiel, Germany; b School of Chemistry, University of Southampton, Highfield, Southampton SO17 1BJ, England

## Abstract

The crystal structure of the title compound, [Cu_2_(C_2_H_4_O_6_PS)(OH)(H_2_O)_2_]·3H_2_O, consists of two Cu^2+^ ions, one (O_3_PC_2_H_4_SO_3_)^3−^ ion and one OH^−^ ion, as well as five water mol­ecules, two of which are coordinated to Cu^2+^. The Cu^2+^ ions are coordinated by six O atoms. The CuO_6_ polyhedra are connected by μ- and μ_3_-O atoms into zigzag chains along the *b* axis. These chains are further connected by –CH_2_CH_2_– groups to form layers, in turn building a three-dimensional framework *via* hydrogen bonding.

## Related literature

For related structures, see: Sonnauer *et al.* (2007 ▶); Sonnauer & Stock (2008*a* ▶,*b* ▶); Benedetto *et al.* (1997 ▶); Adani *et al.* (1998 ▶); Du *et al.* (2006*a* ▶,*b* ▶); Du, Li *et al.* (2007 ▶); Du, Prosvirin & Mao (2007 ▶); Du, Xu *et al.* (2007 ▶).
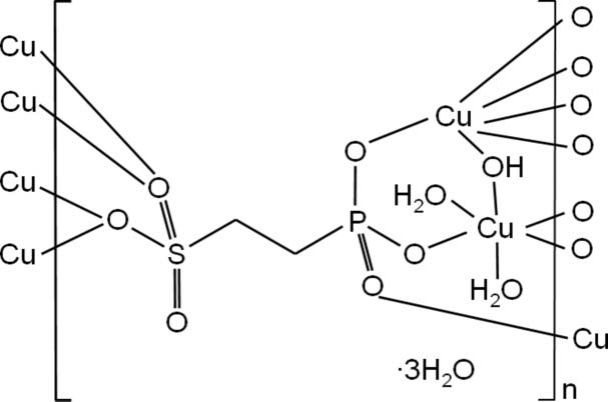


## Experimental

### 

#### Crystal data

[Cu_2_(C_2_H_4_O_6_PS)(OH)(H_2_O)_2_]·3H_2_O
*M* *_r_* = 421.25
Monoclinic, 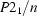

*a* = 10.553 (2) Å
*b* = 7.1312 (14) Å
*c* = 15.791 (3) Å
β = 105.07 (3)°
*V* = 1147.5 (4) Å^3^
*Z* = 4
Mo *K*α radiation
μ = 4.09 mm^−1^
*T* = 120 (2) K
0.16 × 0.05 × 0.02 mm

#### Data collection

Bruker Nonius APEXII CCD diffractometer
Absorption correction: multi-scan (*SADABS*; Sheldrick, 2007 ▶) *T*_min_ = 0.783, *T*_max_ = 0.922
20855 measured reflections
4352 independent reflections
3542 reflections with *I* > 2σ(*I*)
*R*_int_ = 0.061

#### Refinement

*R*[*F* ^2^ > 2σ(*F* ^2^)] = 0.047
*wR*(*F* ^2^) = 0.089
*S* = 1.11
4352 reflections
163 parameters
H-atom parameters constrained
Δρ_max_ = 0.75 e Å^−3^
Δρ_min_ = −0.78 e Å^−3^

### 

Data collection: *COLLECT* (Nonius, 1998 ▶); cell refinement: *DENZO* (Otwinowski & Minor, 1997 ▶) and *COLLECT*; data reduction: *DENZO* and *COLLECT*; program(s) used to solve structure: *SHELXS97* (Sheldrick, 2008 ▶); program(s) used to refine structure: *SHELXL97* (Sheldrick, 2008 ▶); molecular graphics: *DIAMOND* (Brandenburg & Putz, 1999 ▶); software used to prepare material for publication: *publCIF* (Westrip, 2008 ▶).

## Supplementary Material

Crystal structure: contains datablocks I, global. DOI: 10.1107/S1600536808033229/bt2803sup1.cif

Structure factors: contains datablocks I. DOI: 10.1107/S1600536808033229/bt2803Isup2.hkl

Additional supplementary materials:  crystallographic information; 3D view; checkCIF report

## Figures and Tables

**Table 1:** Hydrogen-bond geometry (Å, °)

*D*—H⋯*A*	*D*—H	H⋯*A*	*D*⋯*A*	*D*—H⋯*A*
O*W*1—H2O1⋯O*W*3^i^	0.82	1.90	2.709 (3)	168
O*W*1—H1O1⋯O6^ii^	0.82	2.10	2.802 (3)	144
O*W*2—H1O2⋯O6^ii^	0.82	1.90	2.689 (3)	162
O*W*2—H2O2⋯O*W*4^ii^	0.82	1.85	2.634 (3)	159
O*W*3—H1O3⋯O1	0.82	1.89	2.692 (3)	166
O*W*3—H2O3⋯O*W*5^iii^	0.82	2.16	2.967 (4)	170
O*W*4—H2O4⋯O2^iii^	0.82	1.89	2.707 (3)	174
O*W*4—H1O4⋯O*W*5^iii^	0.82	1.97	2.677 (4)	144
O*W*5—H1O5⋯O*W*2^iv^	0.82	2.32	2.912 (4)	130
O*W*5—H2O5⋯O*W*4^v^	0.82	1.93	2.735 (4)	168

Symmetry codes: (i) 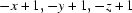; (ii) 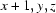; (iii) 
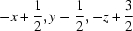
; (iv) 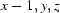; (v) 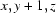.

**Fig. 1.**
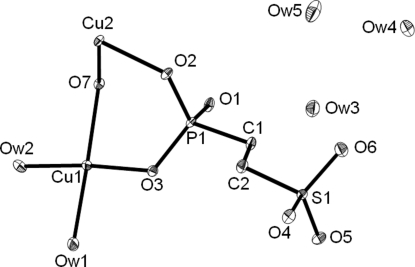
Asymmetric unit of the title compound. Displacement ellipsoids are drawn at the 50% probability level.

**Fig. 2.**
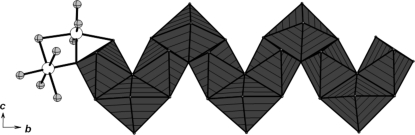
Chains of edge-sharing CuO6 polyhedra along the b-axis. Polyhedra are shaded in grey.

**Fig. 3.**
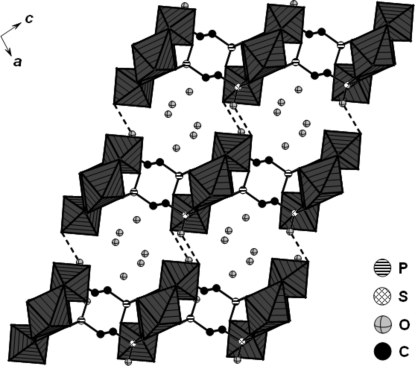
The framework consists of layers, which are connected via hydrogen bonds (dotted line).

## supplementary crystallographic information

### Comment

Inorganic–organic hybrid materials based on metal carboxylates, sulfonates and phosphonates are intensively investigated due to their potential application in the field of gas separation, storage, as well as catalysis, or as sensor materials. We are interested in the use of organic ligands containing two or more different functional groups for the synthesis of functionalized hybrid compounds. Although a large number of metal phosphonates and metal sulfonates have been reported in the literature, compounds based on ligands containing simultaneously a phosphonic as well as a sulfonic acid group have only recently been investigated. These few studies are limited to the use of linker molecules based on rigid phosphonoarylsulfonic acids. Our group has started a systematic investigation using the flexible linker 2-phosphonoethansulfonic acid, which has been reported in the literature (Sonnauer *et al.*, 2007; Sonnauer & Stock, 2008*a*,*b*). Here we report the crystal structure of the new copper phosphonatosulfonate Cu_2_[(O_3_PC_2_H_4_SO_3_)(OH)(H_2_O)_2_](H_2_O)_3_, which was obtained from a hydrothermal reaction in a glass tube.

The title compound consists of two crystallographic independent copper(II) ions, one fully deprotonated (O_3_PC_2_H_4_SO_3_)^3-^ anion, one hydroxide ion, as well as five water molecules (two coordinated to the copper ions)(Fig. 1). The copper ions are coordinated by six oxygen atoms and form CuO_6_ polyhedra. These polyhedra are connected by µ-O and µ_3_-O atoms. Thus, Cu—O—Cu zigzag chains of edge-sharing polyhedra are observed (Fig. 2), which are connected by the organic group –C_2_H_4_– to form layers. These layers are connected *via* hydrogen bonds into a three-dimensional framework (Fig. 3).

### Experimental

H_2_O_3_PC_2_H_4_SO_3_H was synthesized as previously reported (Sonnauer & Stock, 2008*b*). All other reagents were of analytical grade (Aldrich and Fluka) and were used without further purification. The synthesis was performed in a glass reactor (DURAN culture tubes 12 × 100 mm D50 GL 14 M.KAP, SCHOTT 261351155). 263 µl of 2.0 *M* H_3_*L* (0.53 mmol), 536 µl of 2.0 *M* Cu(NO_3_)_2_ (1.06 mmol), and 789 µl of 2.0 *M* NaOH (1.59 mmol) were mixed and H_2_O was added to give the final volume of 2900 µl. The mixture was heated at 90 °C for 24 h. After filtration single-crystals were isolated from the filtrate.

### Refinement

The hydrogen atoms of the C—H groups were positioned with idealized geometry and were refined using a riding model. The hydrogen atoms of the O—H groups were located in the Fourier difference map, their bond lengths were set to ideal values and afterwards the atom positions were refined using a riding model with U(H) = 1.2U_eq_(C) or U(H) = 1.5U_eq_(O).

### Crystal data

**Table:**

[Cu_2_(C_2_H_4_O_6_PS)(OH)(H_2_O)_2_]·3H_2_O	*F*(000) = 848
*M**_r_* = 421.25	*D*_x_ = 2.438 Mg m^−^^3^
Monoclinic, *P*2_1_/*n*	Mo *K*α radiation, λ = 0.71073 Å
Hall symbol: -P 2yn	Cell parameters from 30417 reflections
*a* = 10.553 (2) Å	θ = 2.9–33.1°
*b* = 7.1312 (14) Å	µ = 4.09 mm^−^^1^
*c* = 15.791 (3) Å	*T* = 120 K
β = 105.07 (3)°	Plate, colourless
*V* = 1147.5 (4) Å^3^	0.16 × 0.05 × 0.02 mm
*Z* = 4	

### Data collection

**Table:**

Bruker Nonius APEXII CCD diffractometer	4352 independent reflections
Radiation source: Bruker Nonius FR591 rotating-anode	3542 reflections with *I* > 2σ(*I*)
10cm confocal mirrors	*R*_int_ = 0.061
Detector resolution: 4096 pixels mm^-1^	θ_max_ = 33.1°, θ_min_ = 3.2°
φ and ω scans	*h* = −15→16
Absorption correction: multi-scan (SADABS; Sheldrick, 2007)	*k* = −10→10
*T*_min_ = 0.783, *T*_max_ = 0.922	*l* = −24→24
20855 measured reflections	

### Refinement

**Table:**

Refinement on *F*^2^	Primary atom site location: structure-invariant direct methods
Least-squares matrix: full	Secondary atom site location: difference Fourier map
*R*[*F*^2^ > 2σ(*F*^2^)] = 0.047	Hydrogen site location: inferred from neighbouring sites
*wR*(*F*^2^) = 0.089	H-atom parameters constrained
*S* = 1.11	*w* = 1/[σ^2^(*F*_o_^2^) + 7.2829*P*] where *P* = (*F*_o_^2^ + 2*F*_c_^2^)/3
4352 reflections	(Δ/σ)_max_ < 0.001
163 parameters	Δρ_max_ = 0.75 e Å^−^^3^
0 restraints	Δρ_min_ = −0.78 e Å^−^^3^
0 constraints	

### Special details

**Table:**

Geometry. All e.s.d.'s (except the e.s.d. in the dihedral angle between two l.s. planes) are estimated using the full covariance matrix. The cell e.s.d.'s are taken into account individually in the estimation of e.s.d.'s in distances, angles and torsion angles; correlations between e.s.d.'s in cell parameters are only used when they are defined by crystal symmetry. An approximate (isotropic) treatment of cell e.s.d.'s is used for estimating e.s.d.'s involving l.s. planes.
Refinement. Refinement of *F*^2^ against ALL reflections. The weighted *R*-factor *wR* and goodness of fit *S* are based on *F*^2^, conventional *R*-factors *R* are based on *F*, with *F* set to zero for negative *F*^2^. The threshold expression of *F*^2^ > σ(*F*^2^) is used only for calculating *R*-factors(gt) *etc*. and is not relevant to the choice of reflections for refinement. *R*-factors based on *F*^2^ are statistically about twice as large as those based on *F*, and *R*- factors based on ALL data will be even larger.

### Fractional atomic coordinates and isotropic or equivalent isotropic displacement parameters (Å^2^)

**Table:**

	*x*	*y*	*z*	*U*_iso_*/*U*_eq_	
Cu1	0.82890 (4)	0.68693 (6)	0.59499 (2)	0.00711 (8)	
Cu2	0.75171 (4)	0.93431 (6)	0.75238 (3)	0.00646 (8)	
P1	0.55820 (7)	0.69233 (12)	0.62006 (5)	0.00591 (13)	
O1	0.5805 (2)	0.5079 (3)	0.67215 (15)	0.0087 (4)	
O2	0.5796 (2)	0.8648 (3)	0.68088 (14)	0.0082 (4)	
O3	0.6414 (2)	0.7048 (3)	0.55453 (14)	0.0080 (4)	
S1	0.18276 (7)	0.82114 (11)	0.43204 (5)	0.00632 (12)	
O4	0.1526 (2)	0.9848 (3)	0.37393 (15)	0.0099 (4)	
O5	0.1833 (2)	0.6481 (3)	0.38242 (15)	0.0097 (4)	
O6	0.0958 (2)	0.8096 (4)	0.49057 (15)	0.0123 (4)	
C1	0.3885 (3)	0.6862 (5)	0.56008 (19)	0.0083 (5)	
H1A	0.3725	0.5717	0.5258	0.010*	
H1B	0.3350	0.6820	0.6015	0.010*	
C2	0.3448 (3)	0.8532 (4)	0.4986 (2)	0.0096 (5)	
H2A	0.4044	0.8679	0.4615	0.012*	
H2B	0.3486	0.9667	0.5330	0.012*	
O7	0.8254 (2)	0.6840 (3)	0.72394 (13)	0.0067 (4)	
H7	0.9031	0.6654	0.7482	0.010*	
OW1	0.8352 (2)	0.6968 (4)	0.47036 (15)	0.0138 (5)	
H1O1	0.8932	0.7573	0.4569	0.021*	
H1O2	1.0598	0.7033	0.5919	0.021*	
OW2	1.0209 (2)	0.6627 (3)	0.62678 (14)	0.0106 (4)	
H2O1	0.7633	0.7017	0.4347	0.016*	
H2O2	1.0403	0.5590	0.6487	0.016*	
OW3	0.3840 (2)	0.2617 (4)	0.66291 (16)	0.0153 (5)	
H1O3	0.4395	0.3368	0.6564	0.023*	
H2O3	0.3752	0.3195	0.7059	0.023*	
OW4	0.1112 (2)	0.3811 (4)	0.73327 (16)	0.0147 (5)	
H1O4	0.1639	0.4569	0.7617	0.022*	
H2O4	0.0512	0.3840	0.7574	0.022*	
OW5	0.1476 (3)	1.0144 (4)	0.6947 (2)	0.0227 (6)	
H1O5	0.0800	0.9714	0.6626	0.034*	
H2O5	0.1251	1.1196	0.7059	0.034*	

### Atomic displacement parameters (Å^2^)

**Table:**

	*U*^11^	*U*^22^	*U*^33^	*U*^12^	*U*^13^	*U*^23^
Cu1	0.00682 (16)	0.00862 (17)	0.00601 (15)	−0.00029 (14)	0.00186 (12)	0.00002 (13)
Cu2	0.00614 (15)	0.00526 (15)	0.00695 (15)	−0.00005 (12)	−0.00012 (11)	−0.00153 (12)
P1	0.0060 (3)	0.0054 (3)	0.0054 (3)	−0.0002 (3)	−0.0003 (2)	−0.0005 (3)
O1	0.0066 (10)	0.0079 (10)	0.0096 (10)	−0.0010 (8)	−0.0014 (8)	0.0024 (8)
O2	0.0094 (10)	0.0059 (9)	0.0083 (9)	−0.0011 (8)	0.0001 (8)	−0.0021 (7)
O3	0.0061 (9)	0.0104 (10)	0.0069 (9)	0.0002 (8)	0.0008 (7)	−0.0003 (8)
S1	0.0069 (3)	0.0057 (3)	0.0059 (3)	0.0002 (2)	0.0007 (2)	0.0003 (2)
O4	0.0107 (10)	0.0070 (10)	0.0106 (10)	0.0008 (8)	0.0003 (8)	0.0016 (8)
O5	0.0124 (10)	0.0064 (10)	0.0093 (9)	0.0006 (8)	0.0008 (8)	−0.0013 (8)
O6	0.0105 (10)	0.0144 (11)	0.0122 (10)	0.0006 (9)	0.0036 (8)	0.0017 (9)
C1	0.0067 (12)	0.0080 (12)	0.0090 (11)	−0.0011 (11)	−0.0002 (9)	0.0005 (11)
C2	0.0095 (13)	0.0085 (13)	0.0099 (12)	−0.0014 (10)	0.0007 (10)	0.0008 (10)
O7	0.0069 (9)	0.0050 (9)	0.0077 (8)	0.0004 (8)	0.0010 (7)	0.0000 (8)
OW1	0.0091 (10)	0.0230 (13)	0.0085 (9)	−0.0046 (10)	0.0010 (8)	0.0012 (9)
OW2	0.0121 (10)	0.0114 (11)	0.0102 (9)	0.0001 (8)	0.0061 (8)	0.0016 (8)
OW3	0.0141 (11)	0.0161 (12)	0.0146 (11)	−0.0034 (9)	0.0019 (9)	0.0018 (9)
OW4	0.0126 (11)	0.0165 (12)	0.0164 (11)	0.0000 (9)	0.0063 (9)	0.0016 (9)
OW5	0.0161 (13)	0.0183 (13)	0.0310 (15)	−0.0033 (11)	0.0010 (11)	−0.0078 (12)

### Geometric parameters (Å, °)

**Table:**

Cu1—O3	1.919 (2)	O4—Cu1^i^	2.389 (2)
Cu1—OW2	1.965 (2)	O5—Cu2^vi^	2.420 (2)
Cu1—OW1	1.988 (2)	O5—Cu1^ii^	2.424 (2)
Cu1—O7	2.046 (2)	C1—C2	1.530 (4)
Cu1—O4^i^	2.389 (2)	C1—H1A	0.9700
Cu1—O5^ii^	2.424 (2)	C1—H1B	0.9700
Cu2—O1^iii^	1.932 (2)	C2—H2A	0.9700
Cu2—O2	1.937 (2)	C2—H2B	0.9700
Cu2—O7^iii^	2.033 (2)	O7—Cu2^v^	2.033 (2)
Cu2—O7	2.043 (2)	O7—H7	0.8200
Cu2—O5^iv^	2.420 (2)	OW1—H1O1	0.8199
P1—O3	1.524 (2)	OW1—H2O1	0.8200
P1—O1	1.537 (2)	OW2—H1O2	0.8199
P1—O1	1.537 (2)	OW2—H2O2	0.8200
P1—O2	1.541 (2)	OW3—H1O3	0.8200
P1—C1	1.795 (3)	OW3—H2O3	0.8200
O1—Cu2^v^	1.932 (2)	OW4—H1O4	0.8200
S1—O5	1.463 (2)	OW4—H2O4	0.8200
S1—O6	1.465 (2)	OW5—H1O5	0.8199
S1—O4	1.468 (2)	OW5—H2O5	0.8200
S1—C2	1.774 (3)		
			
O3—Cu1—OW2	175.39 (9)	O5—S1—O6	112.38 (15)
O3—Cu1—OW1	88.01 (10)	O5—S1—O4	111.51 (13)
OW2—Cu1—OW1	87.60 (10)	O6—S1—O4	111.70 (14)
O3—Cu1—O7	92.77 (9)	O5—S1—C2	106.77 (15)
OW2—Cu1—O7	91.66 (9)	O6—S1—C2	107.42 (14)
OW1—Cu1—O7	178.32 (10)	O4—S1—C2	106.68 (14)
O3—Cu1—O4^i^	91.45 (9)	S1—O4—Cu1^i^	131.14 (14)
OW2—Cu1—O4^i^	90.56 (9)	S1—O5—Cu2^vi^	134.67 (14)
OW1—Cu1—O4^i^	98.45 (10)	S1—O5—Cu1^ii^	138.17 (14)
O7—Cu1—O4^i^	80.05 (9)	Cu2^vi^—O5—Cu1^ii^	85.68 (7)
O3—Cu1—O5^ii^	91.43 (9)	C2—C1—P1	114.3 (2)
OW2—Cu1—O5^ii^	88.08 (9)	C2—C1—H1A	108.7
OW1—Cu1—O5^ii^	101.37 (10)	P1—C1—H1A	108.7
O7—Cu1—O5^ii^	80.11 (8)	C2—C1—H1B	108.7
O4^i^—Cu1—O5^ii^	160.06 (8)	P1—C1—H1B	108.7
O1^iii^—Cu2—O2	177.30 (10)	H1A—C1—H1B	107.6
O1^iii^—Cu2—O7^iii^	89.73 (9)	C1—C2—S1	111.2 (2)
O2—Cu2—O7^iii^	88.38 (9)	C1—C2—H2A	109.4
O1^iii^—Cu2—O7	91.86 (9)	S1—C2—H2A	109.4
O2—Cu2—O7	90.07 (9)	C1—C2—H2B	109.4
O7^iii^—Cu2—O7	177.92 (2)	S1—C2—H2B	109.4
O1^iii^—Cu2—O5^iv^	88.29 (9)	H2A—C2—H2B	108.0
O2—Cu2—O5^iv^	89.48 (9)	Cu2^v^—O7—Cu2	122.09 (10)
O7^iii^—Cu2—O5^iv^	80.47 (8)	Cu2^v^—O7—Cu1	107.67 (10)
O7—Cu2—O5^iv^	100.90 (8)	Cu2—O7—Cu1	108.43 (10)
O3—P1—O1	112.24 (13)	Cu2^v^—O7—H7	99.9
O3—P1—O1	112.24 (13)	Cu2—O7—H7	115.5
O3—P1—O2	111.09 (13)	Cu1—O7—H7	101.1
O1—P1—O2	111.86 (12)	Cu1—OW1—H1O1	119.6
O1—P1—O2	111.86 (12)	Cu1—OW1—H2O1	114.7
O3—P1—C1	108.35 (13)	H1O1—OW1—H2O1	114.8
O1—P1—C1	104.75 (14)	Cu1—OW2—H1O2	117.4
O1—P1—C1	104.75 (14)	Cu1—OW2—H2O2	108.3
O2—P1—C1	108.21 (14)	H1O2—OW2—H2O2	119.3
P1—O1—Cu2^v^	123.42 (14)	H1O3—OW3—H2O3	90.8
P1—O2—Cu2	122.06 (14)	H1O4—OW4—H2O4	103.0
P1—O3—Cu1	119.75 (13)	H1O5—OW5—H2O5	102.8

Symmetry codes: (i) −*x*+1, −*y*+2, −*z*+1; (ii) −*x*+1, −*y*+1, −*z*+1; (iii) −*x*+3/2, *y*+1/2, −*z*+3/2; (iv) *x*+1/2, −*y*+3/2, *z*+1/2;  (v) −*x*+3/2, *y*−1/2, −*z*+3/2; (vi) *x*−1/2, −*y*+3/2, *z*−1/2.

### Hydrogen-bond geometry (Å, °)

**Table:**

*D*—H···*A*	*D*—H	H···*A*	*D*···*A*	*D*—H···*A*
OW1—H2O1···OW3^ii^	0.82	1.90	2.709 (3)	168
OW1—H1O1···O6^vii^	0.82	2.10	2.802 (3)	144
OW2—H1O2···O6^vii^	0.82	1.90	2.689 (3)	162
OW2—H2O2···OW4^vii^	0.82	1.85	2.634 (3)	159
OW3—H1O3···O1	0.82	1.89	2.692 (3)	166
OW3—H2O3···OW5^viii^	0.82	2.16	2.967 (4)	170
OW4—H2O4···O2^viii^	0.82	1.89	2.707 (3)	174
OW4—H1O4···OW5^viii^	0.82	1.97	2.677 (4)	144
OW5—H1O5···OW2^ix^	0.82	2.32	2.912 (4)	130
OW5—H2O5···OW4^x^	0.82	1.93	2.735 (4)	168

Symmetry codes: (ii) −*x*+1, −*y*+1, −*z*+1; (vii) *x*+1, *y*, *z*;  (viii) −*x*+1/2, *y*−1/2, −*z*+3/2; (ix) *x*−1, *y*, *z*; (x) *x*, *y*+1, *z*.
